# Supplementary material for: Transcriptome Assembly at Single-Cell Resolution with Beaver
Source: bioRxiv. 2024 Nov 6:2024.11.04.621958. Preprint. [Version 1] doi: 10.1101/2024.11.04.621958 (PMC11580954; doi:10.1101/2024.11.04.621958)
Supplement: Supplement 1 [file media-1.pdf]

Supplementary Materials for
"Transcriptome Assembly at Single-Cell Resolution with Beaver"

Qian Shi[1], Qimin Zhang[1], and Mingfu Shao[1,2,*]

[1]Department of Computer Science and Engineering, School of Electronic Engineering and Computer Science, The Pennsylvania State University

[2]Huck Institutes of the Life Sciences, The Pennsylvania State University

October 25, 2024

List of Supplementary Notes

1 PseudoCode for Candidate Transcript Generation 3
2 Features Description 4

List of Supplementary Figures

1 Beaver vs. TransMeta on HEK293T dataset. 7
2 Beaver vs. PsiCLASS on HEK293T dataset. 7
3 Beaver vs. Aletsch on HEK293T dataset. 8
4 Beaver vs. StringTie2 on HEK293T dataset. 8
5 Beaver vs. Scallop2 on HEK293T dataset. 9
6 Beaver vs. TransMeta on Mouse-Fibroblast dataset. 9
7 Beaver vs. PsiCLASS on Mouse-Fibroblast dataset. 10
8 Beaver vs. Aletsch on Mouse-Fibroblast dataset. 10
9 Beaver vs. StringTie2 on Mouse-Fibroblast dataset. 11
10 Beaver vs. Scallop2 on Mouse-Fibroblast dataset. 11
11 Beaver vs. TransMeta on HEK293T-Sim dataset. 12

*Correspondence should be addressed to mxs2589@psu.edu.

12 Beaver vs. PsiCLASS on HEK293T-Sim dataset. 12

13 Beaver vs. Aletsch on HEK293T-Sim dataset. 13

14 Beaver vs. StringTie2 on HEK293T-Sim dataset. 13

15 Beaver vs. Scallop2 on HEK293T-Sim dataset. 14

16 Beaver vs. TransMeta on Mouse-Fibroblast-Sim dataset. 14

17 Beaver vs. PsiCLASS on Mouse-Fibroblast-Sim dataset. 15

18 Beaver vs. Aletsch on Mouse-Fibroblast-Sim dataset. 15

19 Beaver vs. StringTie2 on Mouse-Fibroblast-Sim dataset. 16

20 Beaver vs. Scallop2 on Mouse-Fibroblast-Sim dataset. 16

Supplementary Note 1: PseudoCode for Transcript Generation

Algorithm 1 Dynamic Programming for Candidate Transcript Generation

Input: Connected component $G_c = (V_c, E_c)$ ▷ Connected components n transcript fragment graph
Output: Set of optimal paths P_c ▷ Each path represents a candidate transcript

function FINDOPTIMALPATHS(G_c)
    $(v_1, v_2, \ldots, v_{|V_c|}) \leftarrow$ TopologicalSort(G_c) ▷ $O(|V_c| + |E_c|)$
    paths[v] $\leftarrow$ new MinHeap() for all $v \in V_c$ ▷ Initialize Min-Heaps
    for $j \leftarrow 1$ to $|V_c|$ **do**
        paths[v_i].push{(MergingScore(v_i), v_i} ▷ Initialize single-vertex path
        for $(v_i, v_j) \in E_c$ **do** ▷ Check incoming edges to v_j
            for each path p in paths[v_i].getAll() **do** ▷ $O(p_n)$
                new_path $\leftarrow p \cup \{v_j\}$ ▷ Extend path with new vertex
                score $\leftarrow$ MergingScore(new_path)
                if paths[v_j].size() $< p_n$ **then** ▷ Limit p_n paths stored per node
                    paths[v_j].push(score, new_path)
                else if score $>$ paths[v_j].top().score **then** ▷ Heap is full
                    paths[v_j].pop() ▷ Remove lowest scoring path in the Min-Heap
                    paths[v_j].push(score, new_path)
                end if
            end for
        end for
    end for
    return $P_c \leftarrow \text{TopK}(\bigcup_{v \in V_c} \{p \mid (\text{score}, p) \in \text{paths}[v].\text{getAll}()\}, p_c)$ ▷ Limit p_c paths stored per graph
end function
function MERGINGSCORE(path p)
    $b \leftarrow \min_{j \in J(p)}$ JunctionWeight(j, p) ▷ Bottleneck weight
    $n \leftarrow |J(p)|$ ▷ Number of junctions
    return $b \cdot n$
end function
function JUNCTIONWEIGHT(junction j, path p)
    $X_j \leftarrow \{t \mid t$ contains j and is compatible with $p\}$ ▷ Find compatible transcripts
    return $\sum_{t \in X_j}$ coverage(t) ▷ Sum coverage of supporting transcripts
end function

Supplementary Note 2: Features Description

This note describes the feature sets used to train Beaver-General and Beaver-Specific. We developed two feature sets: (1) general features (n=30) that assess overall transcript reliability across the dataset, and (2) cell-specific features (n=21) that evaluate expression likelihood in individual cells. These features are computed for every unique pair of candidate path p and cell c in the transcript fragment graphs, with general features being replicated across all cells for the same p.

(Shown in *Italic* are the headers of the Beaver's feature table.)

General Features ($n = 30$)

- *sample_size*: Total number of input cells/samples.
- *bottleneck_coverage*: Bottleneck junction-score $BJ(p)$ from the dynamic programming solution.
- *highest_coverage*: Maximum coverage value observed among raw transcript fragments $t \sim p$.
- *num_junctions*: Total number of junctions in path p, i.e. $NJ(p)$.
- Compatible junction coverage:

  (*min_comp_cov*, *max_comp_cov*, *median_comp_cov*, *mean_comp_cov*, *std_comp_cov*)

  Statistics of junction-scores defined in Section 2.3, which is the sum of scores of all transcript fragments that contain junction j and are compatible with p, i.e., $J(j,p) := \sum_{t: j \in t \text{ and } t \sim p} score(t)$.
- Junction coverage statistics:

  (*min_junc_cov*, *max_junc_cov*, *median_junc_cov*, *mean_junc_cov*, *std_junc_cov*)

  Statistics of junction-scores regardless of compatibility, i.e., $J'(j,p) := \sum_{t: j \in t} score(t)$.
- *extendable_score*: Cumulative junction scores for potential left/right path extensions.
- *cells_support*: Number of cells supporting path p.
- *ratio_cells_support*: Proportion of total cells supporting path p.
- *ratio_cells_fl_over_sample*: Proportion of cells supporting all junctions in p.

- *ratio_cells_fl_over_cells_support*: Proportion of full-length support among supporting cells.

- Cell support statistics:

  (*min_cell_junc*, *max_cell_junc*, *median_cell_junc*, *mean_cell_junc*, *std_cell_junc*)

  Statistics describing the number of cells supporting individual junctions $j \in p$.

- *num_fragments*: Total number of fragments compatible with p.

- Fragment Connecting:

  (*min_frag_cov*, *max_frag_cov*, *median_frag_cov*, *mean_frag_cov*, *std_frag_cov*)

  Statistics describing fragment coverage along path p.

Cell-Specific Features ($n = 21$)

- *input_genes*: Estimated number of input gene loci in cell c.

- *input_transcripts*: Number of input transcripts in cell c.

- *output_transcripts*: Number of output transcripts in cell c.

- *cell_supporting_junctions*: Number of junctions $j \in p$ supported by c.

- *cell_ratio_supporting_junctions*: Proportion of junctions $j \in p$ is supported by c.

- Cell-specific compatible junction coverage:

  (*cell_min_comp_cov*, *cell_max_comp_cov*, *cell_median_comp_cov*, *cell_mean_comp_cov*, *cell_std_comp_cov*)

  Statistics of cell-specific junction-scores, i.e., $J(c, p, j) := \sum_{t:j \in t \text{ and } t \sim p \text{ and } t \in c} score(t)$.

- *cell_nonzero_comp_cov*: Number of junction $j \in p$ such that $J(c, p, j) \neq 0$.

- *cell_max_streak_comp_cov*: Maximum number of continuous non-zero $J(c, p, j)$ for all junctions $j \in p$.

- *cell_min_streak_comp_cov*:Minimum number of continuous non-zero $J(c, p, j)$ for all junctions $j \in p$.

- Cell-specific Junction coverage:

  (*cell_min_junc_cov*, *cell_max_junc_cov*, *cell_median_junc_cov*, *cell_mean_junc_cov*, *cell_std_junc_cov*)

Statistics of cell-specific junction-scores regardless of compatibility, i.e., $J'(c,p,j) := \sum_{t: j \in t \text{ and } t \in c} score(t)$.

- *cell_nonzero_junc_cov*: Number of junction $j \in p$ such that $J'(c,p,j) \neq 0$.
- *cell_max_streak_junc_cov*: Maximum number of continuous non-zero $J'(c,p,j)$ for all junctions $j \in p$.
- *cell_min_streak_junc_cov*: Minimum number of continuous non-zero $J'(c,p,j)$ for all junctions $j \in p$.

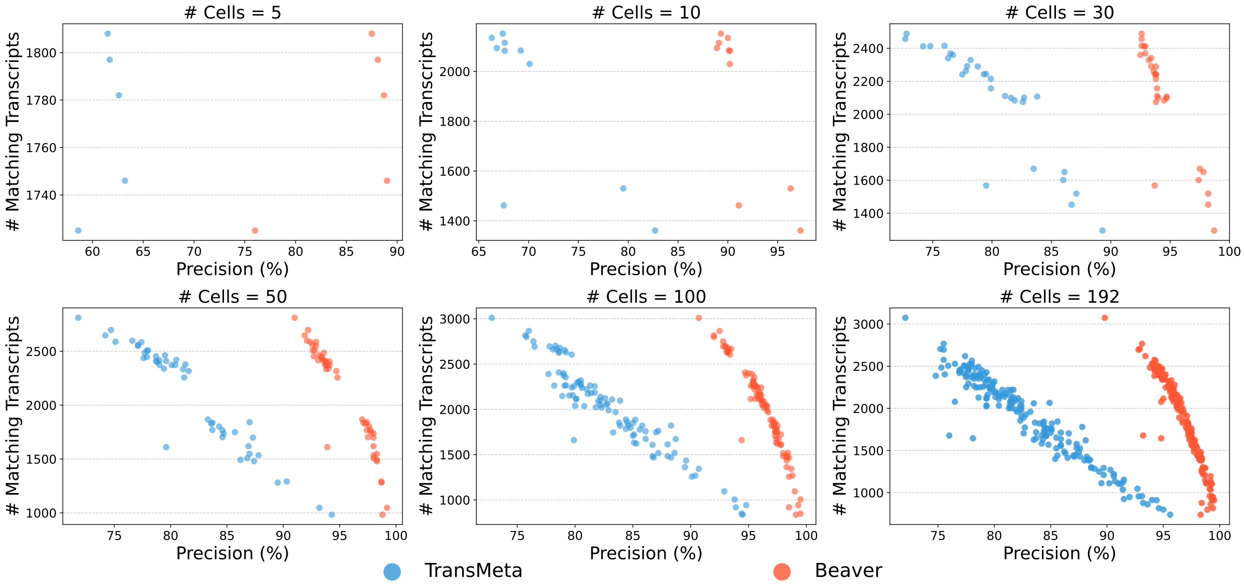

Supplementary Figure 1: Comparison of adjusted precision between Beaver and TransMeta across varying cell population sizes ($n = 5, 10, 30, 50, 100, 192$) in the HEK293T dataset. Each data point represents an individual cell.

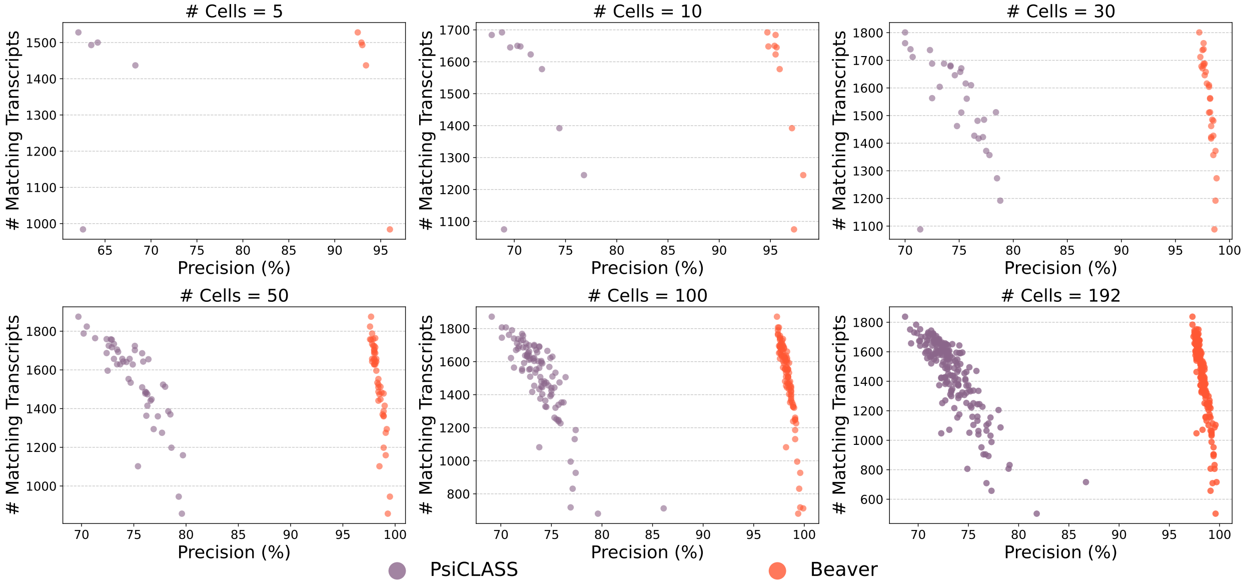

Supplementary Figure 2: Comparison of adjusted precision between Beaver and PsiCLASS across varying cell population sizes ($n = 5, 10, 30, 50, 100, 192$) in the HEK293T dataset. Each data point represents an individual cell.

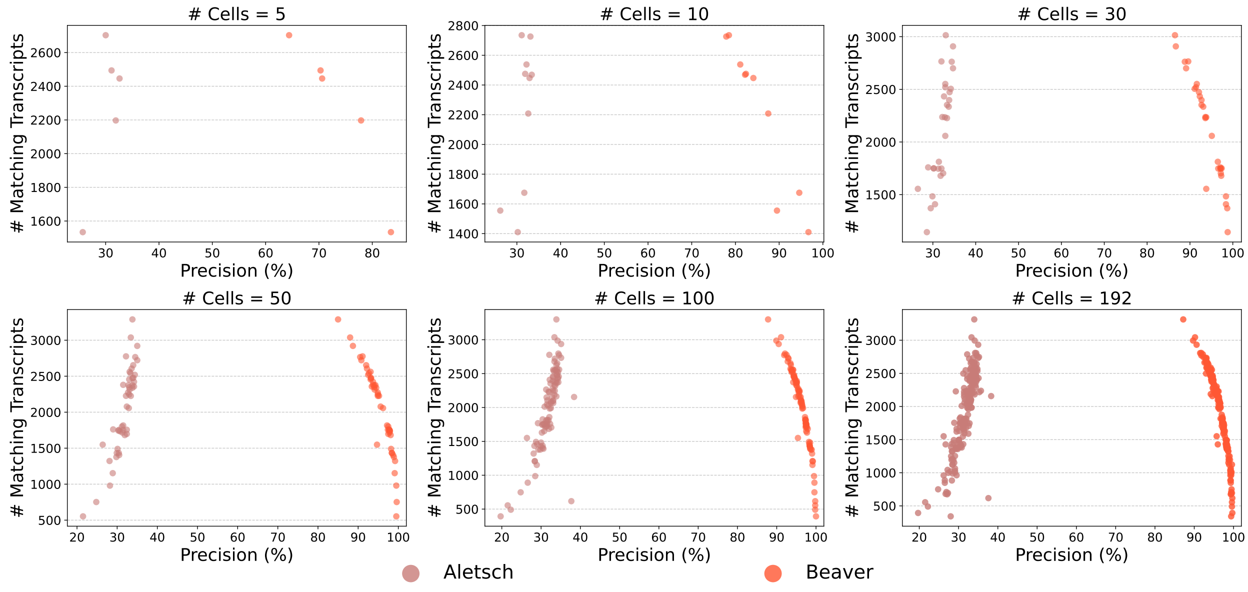

Supplementary Figure 3: Comparison of adjusted precision between Beaver and Aletsch across varying cell population sizes ($n = 5, 10, 30, 50, 100, 192$) in the HEK293T dataset. Each data point represents an individual cell.

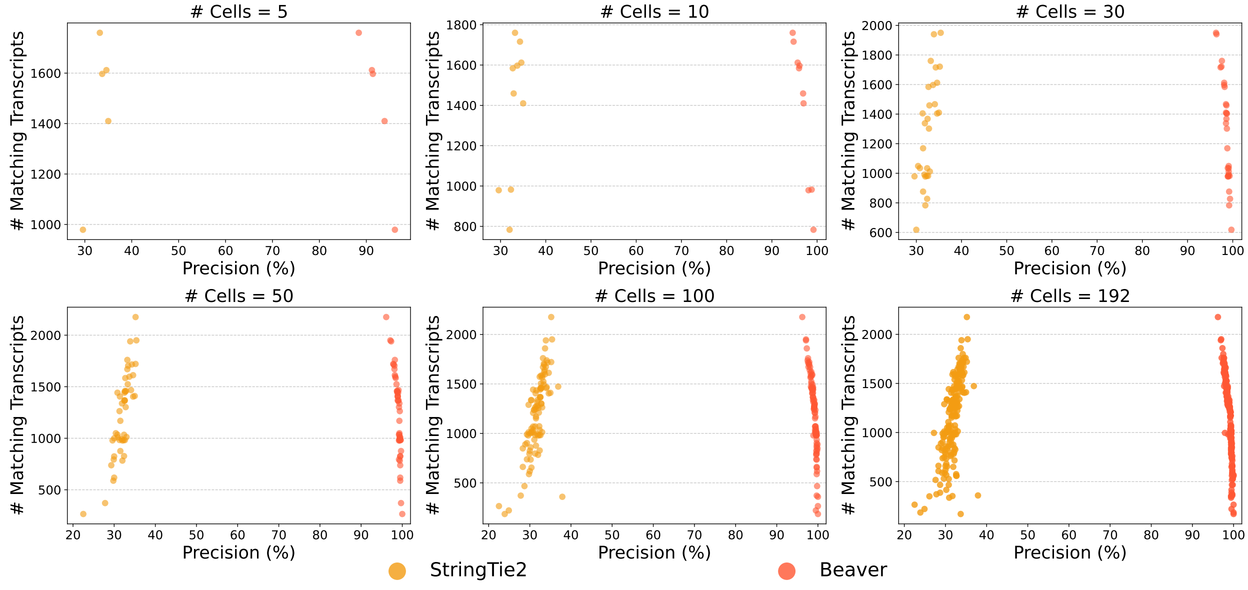

Supplementary Figure 4: Comparison of adjusted precision between Beaver and StringTie2 across varying cell population sizes ($n = 5, 10, 30, 50, 100, 192$) in the HEK293T dataset. Each data point represents an individual cell.

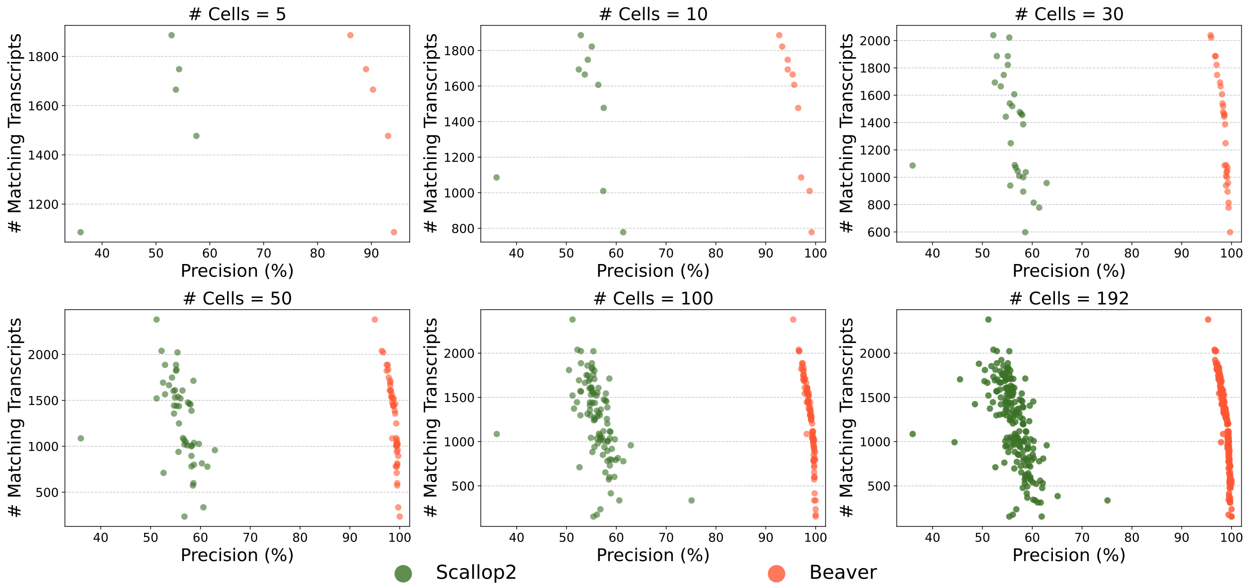

Supplementary Figure 5: Comparison of adjusted precision between Beaver and Scallop2 across varying cell population sizes ($n = 5, 10, 30, 50, 100, 192$) in the HEK293T dataset. Each data point represents an individual cell.

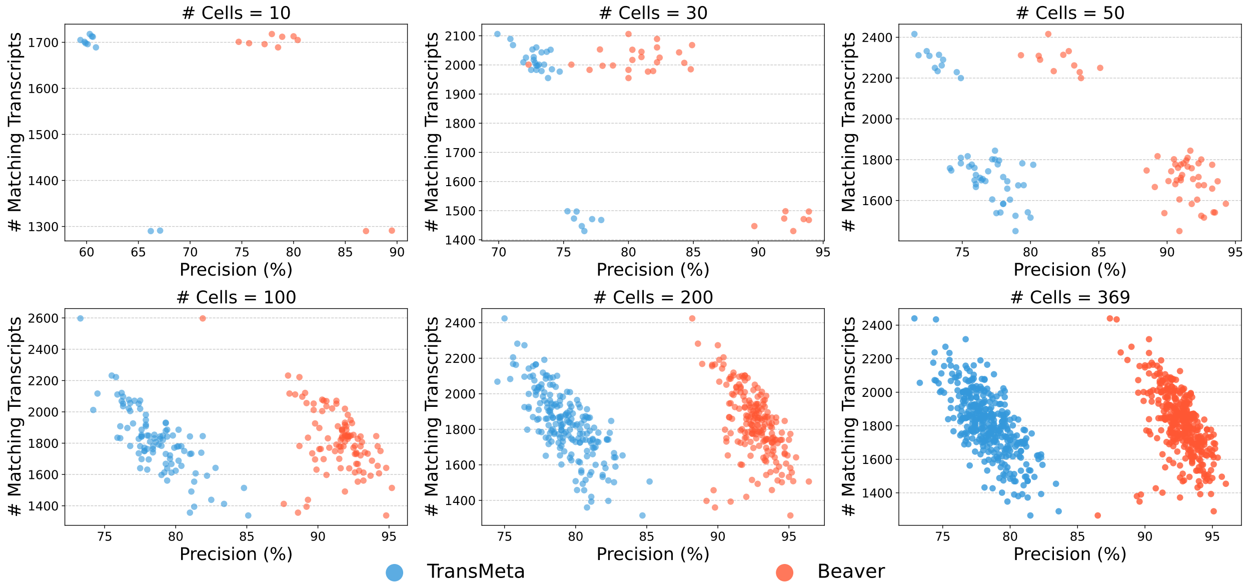

Supplementary Figure 6: Comparison of adjusted precision between Beaver and TransMeta across varying cell population sizes ($n = 10, 30, 50, 100, 200, 369$) in the Mouse-Fibroblast dataset. Each data point represents an individual cell.

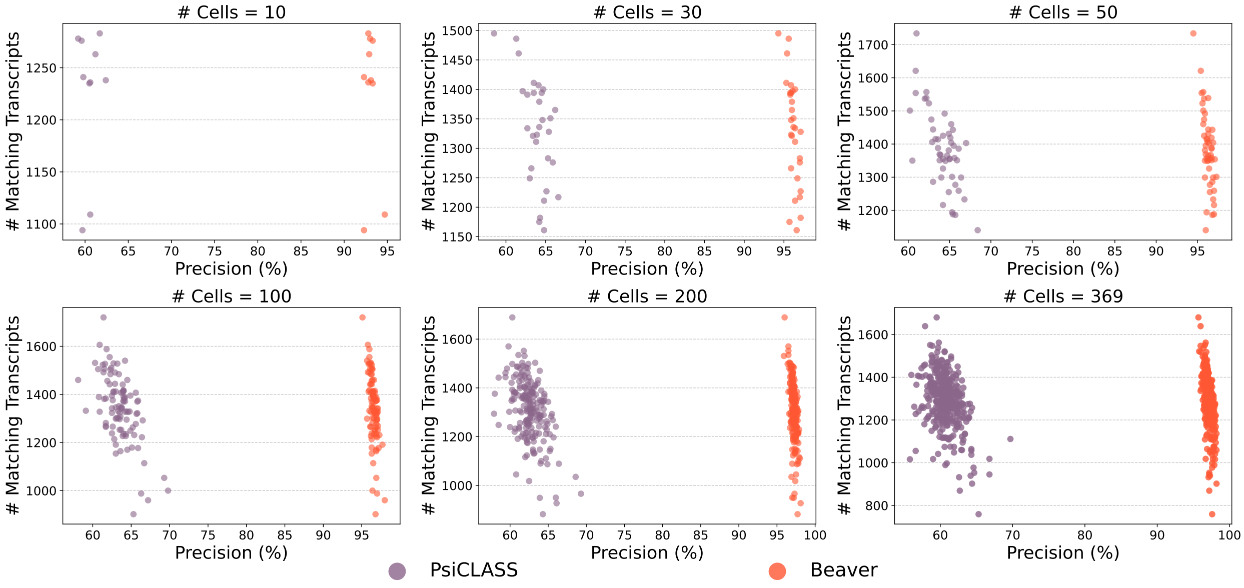

Supplementary Figure 7: Comparison of adjusted precision between Beaver and PsiCLASS across varying cell population sizes ($n = 10, 30, 50, 100, 200, 369$) in the Mouse-Fibroblast dataset. Each data point represents an individual cell.

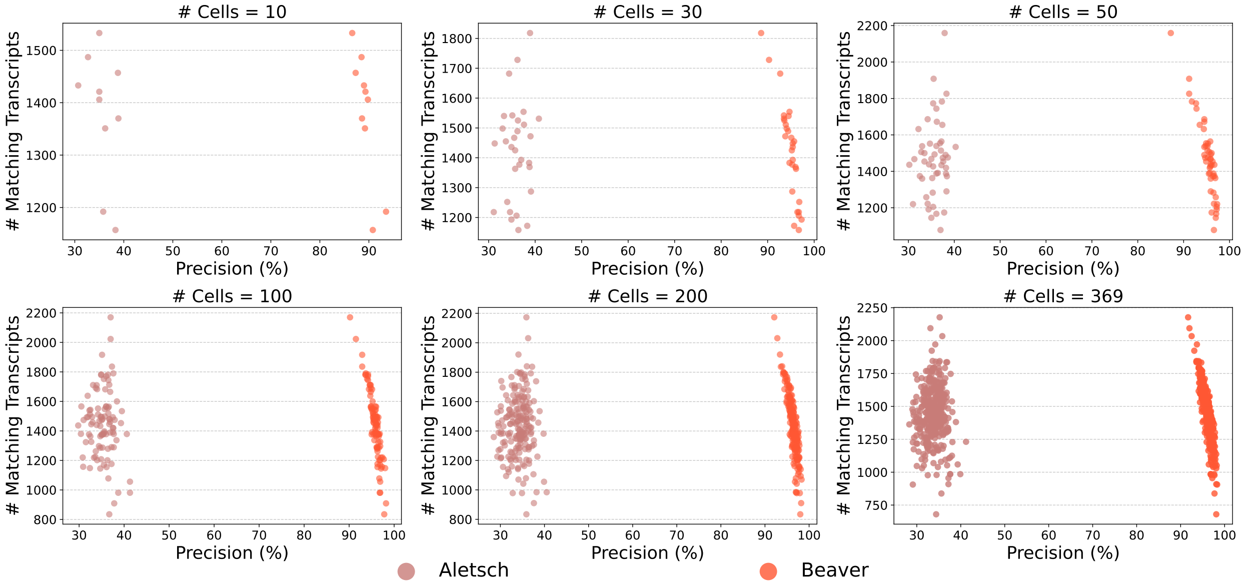

Supplementary Figure 8: Comparison of adjusted precision between Beaver and Aletsch across varying cell population sizes ($n = 10, 30, 50, 100, 200, 369$) in the Mouse-Fibroblast dataset. Each data point represents an individual cell.

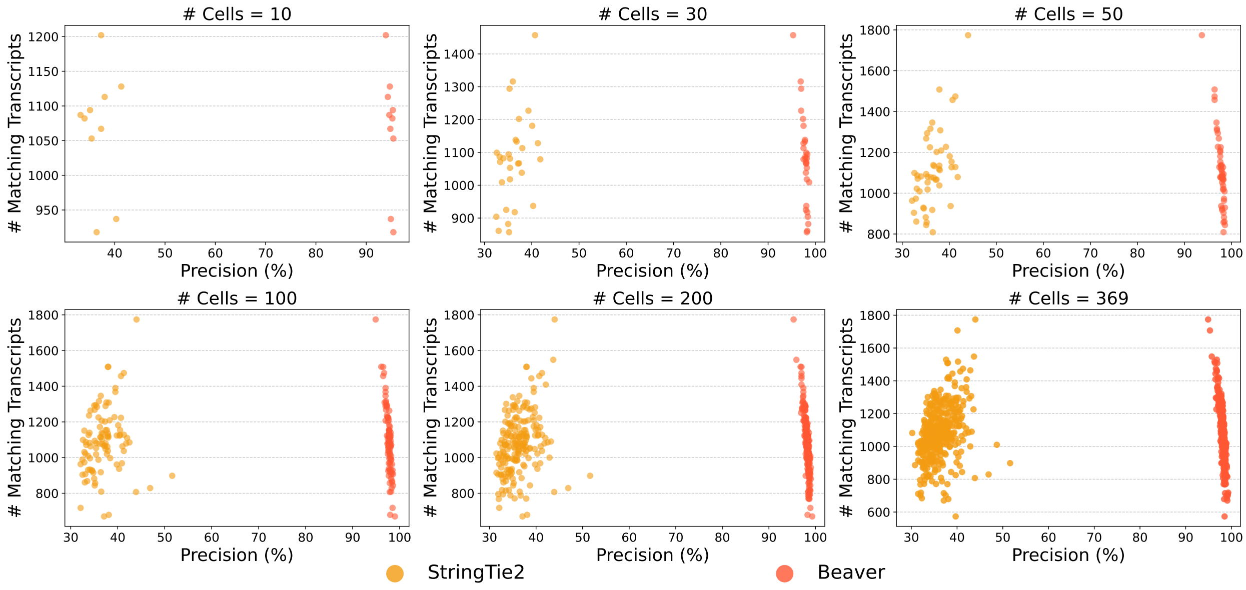

Supplementary Figure 9: Comparison of adjusted precision between Beaver and StringTie2 across varying cell population sizes ($n = 10, 30, 50, 100, 200, 369$) in the Mouse-Fibroblast dataset. Each data point represents an individual cell.

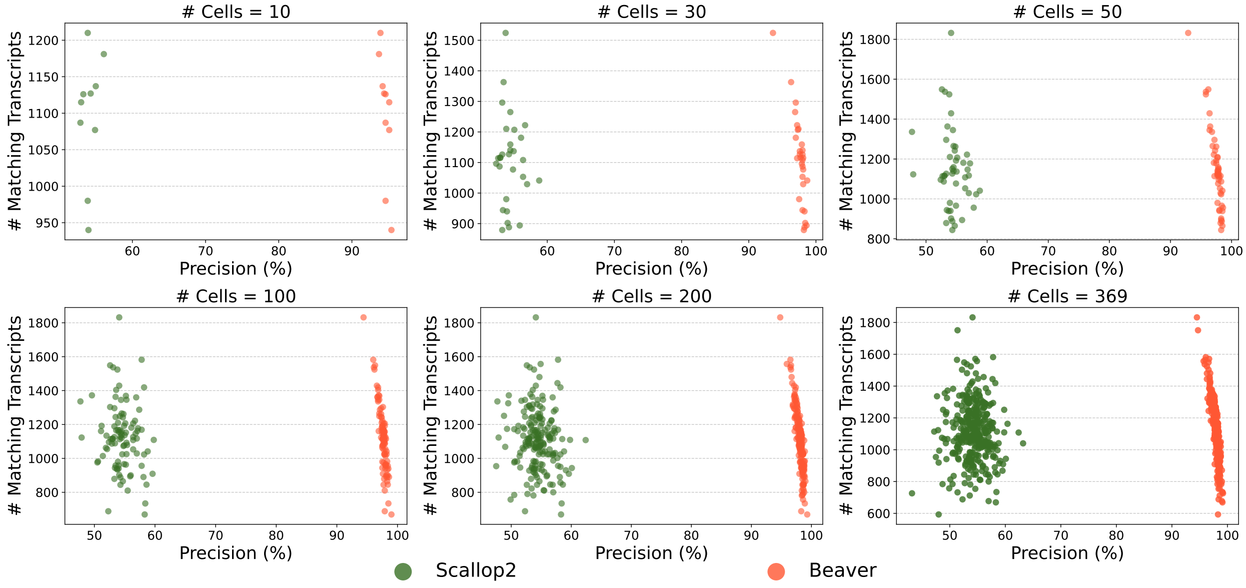

Supplementary Figure 10: Comparison of adjusted precision between Beaver and Scallop2 across varying cell population sizes ($n = 10, 30, 50, 100, 200, 369$) in the Mouse-Fibroblast dataset. Each data point represents an individual cell.

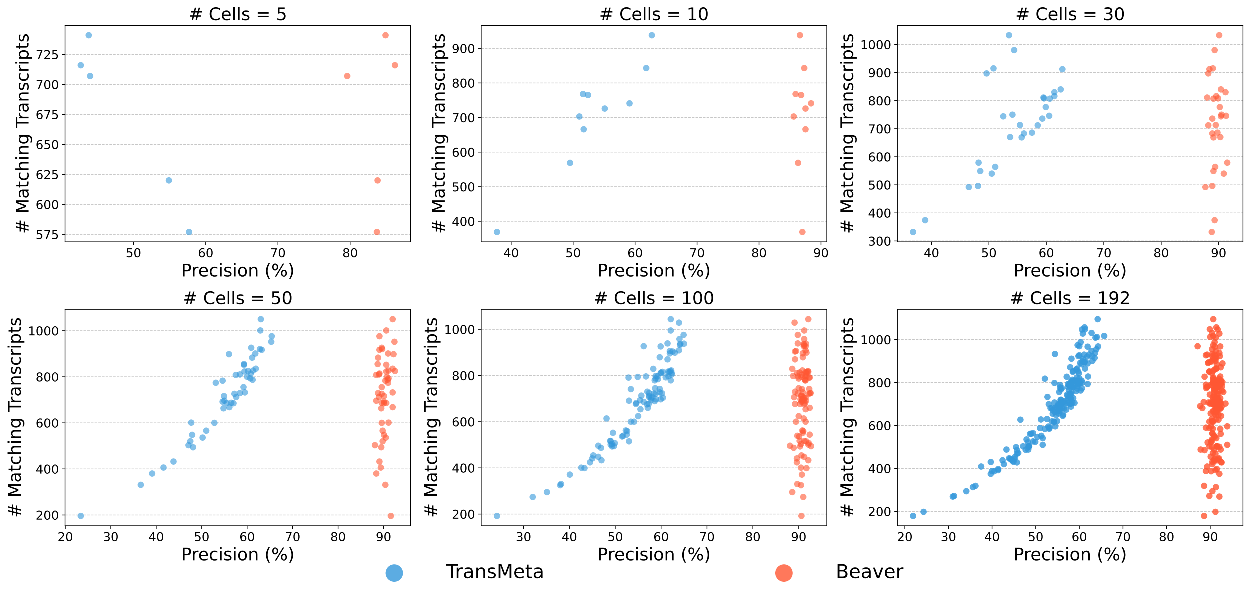

Supplementary Figure 11: Comparison of adjusted precision between Beaver and TransMeta across varying cell population sizes ($n = 5, 10, 30, 50, 100, 192$) in the simulated HEK293T-Sim dataset. Each data point represents an individual cell.

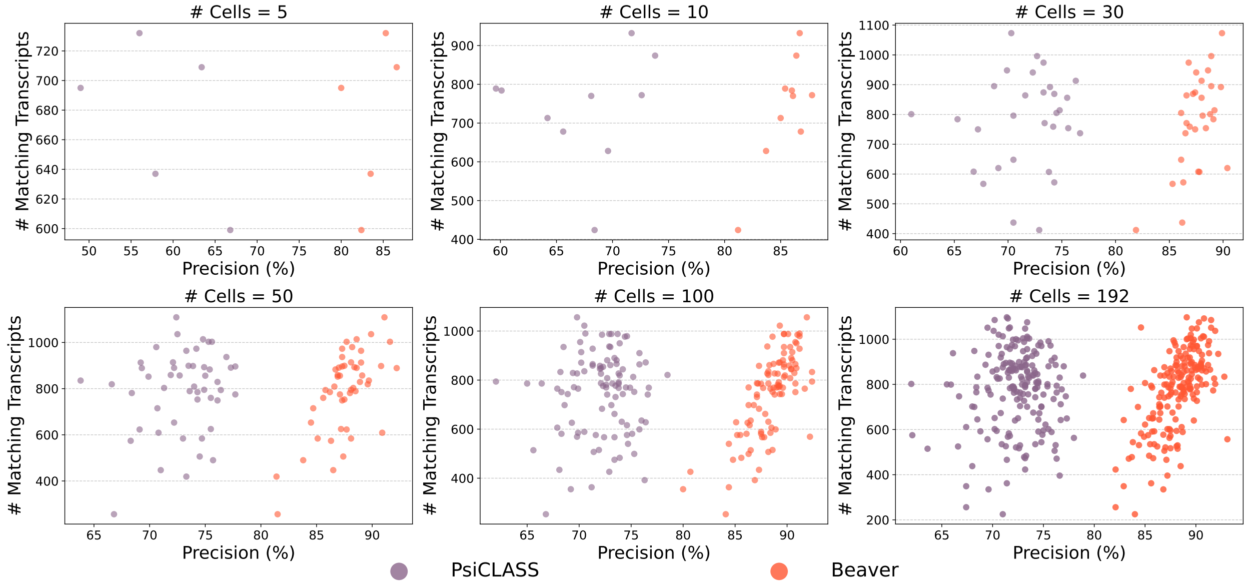

Supplementary Figure 12: Comparison of adjusted precision between Beaver and PsiCLASS across varying cell population sizes ($n = 5, 10, 30, 50, 100, 192$) in the simulated HEK293T-Sim dataset. Each data point represents an individual cell.

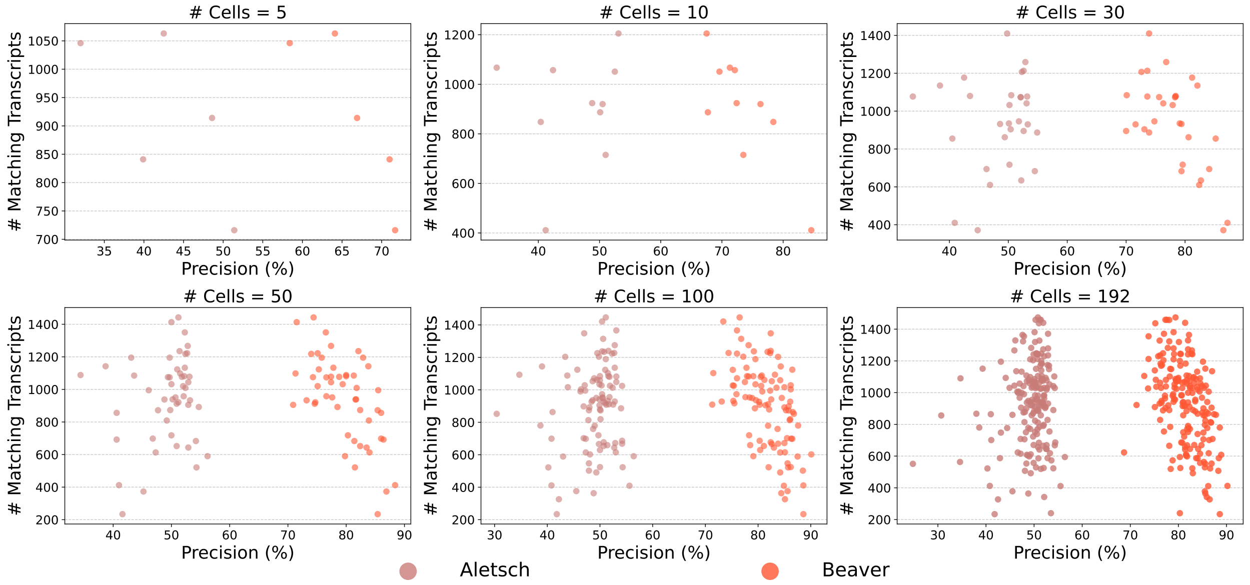

Supplementary Figure 13: Comparison of adjusted precision between Beaver and Aletsch across varying cell population sizes ($n = 5, 10, 30, 50, 100, 192$) in the simulated HEK293T-Sim dataset. Each data point represents an individual cell.

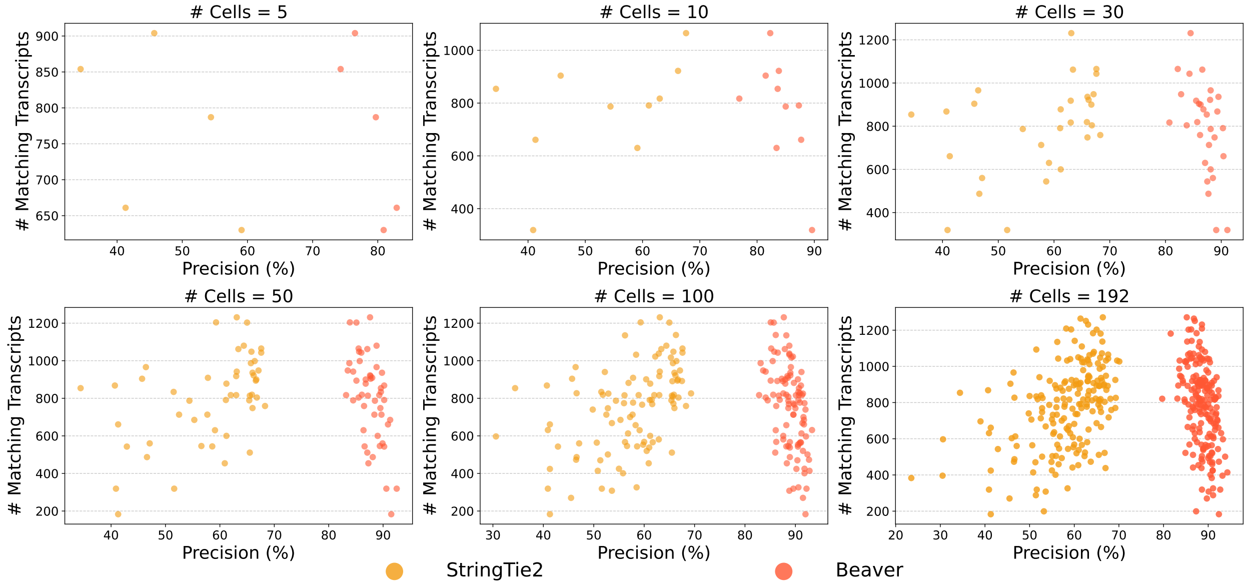

Supplementary Figure 14: Comparison of adjusted precision between Beaver and StringTie2 across varying cell population sizes ($n = 5, 10, 30, 50, 100, 192$) in the simulated HEK293T-Sim dataset. Each data point represents an individual cell.

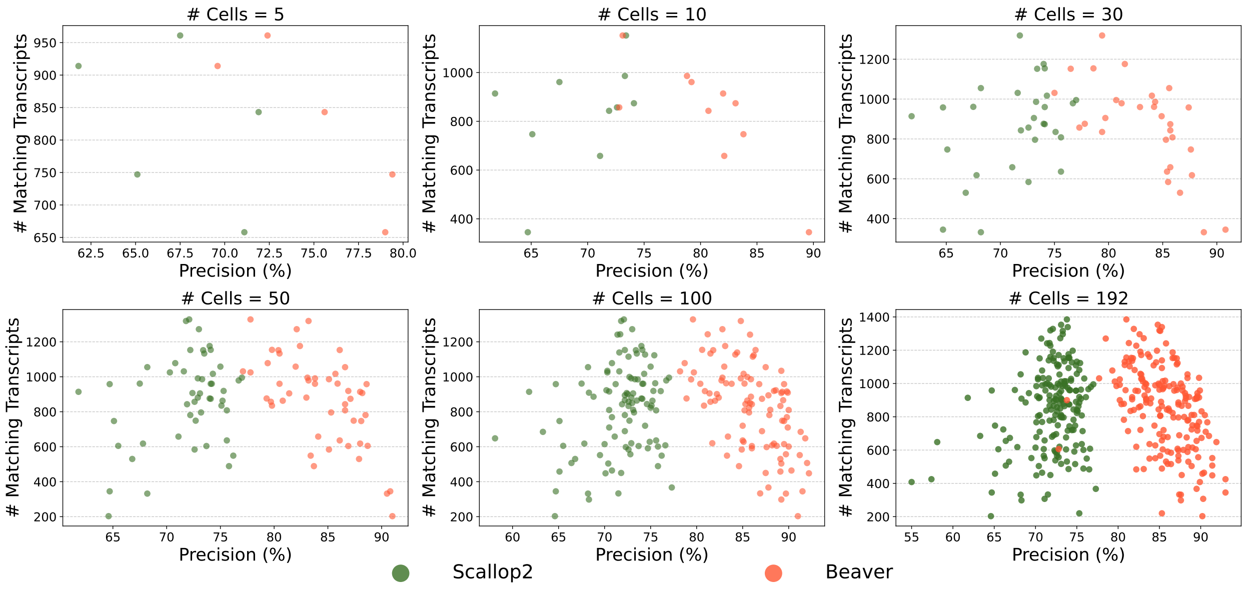

Supplementary Figure 15: Comparison of adjusted precision between Beaver and Scallop2 across varying cell population sizes ($n = 5, 10, 30, 50, 100, 192$) in the simulated HEK293T-Sim dataset. Each data point represents an individual cell.

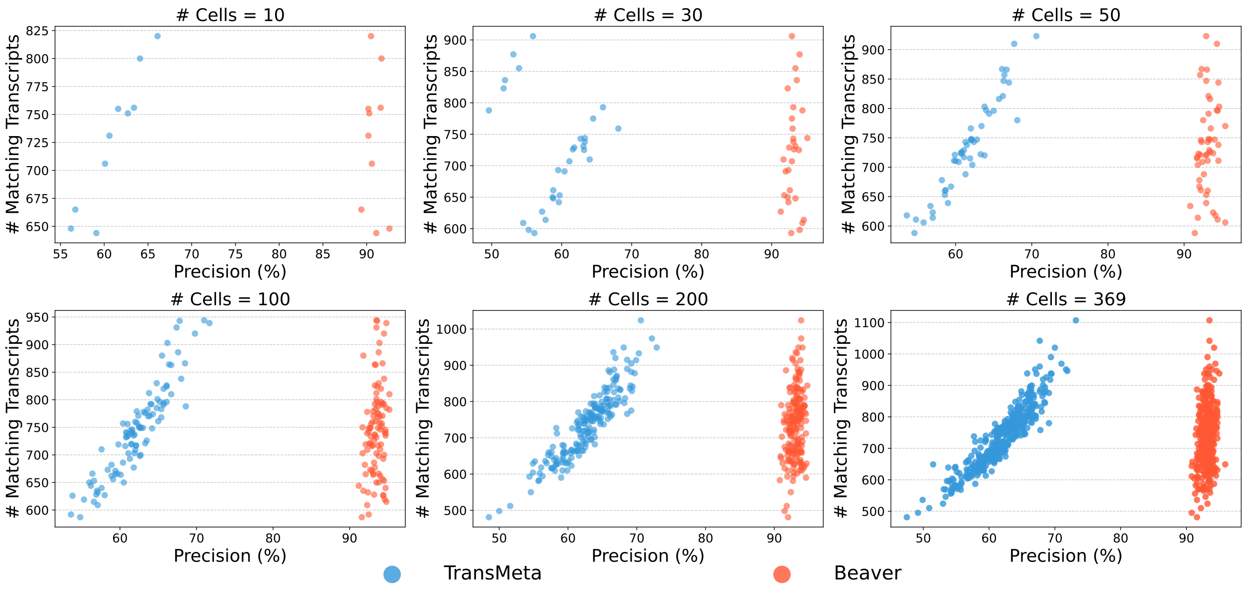

Supplementary Figure 16: Comparison of adjusted precision between Beaver and TransMeta across varying cell population sizes ($n = 10, 30, 50, 100, 200, 369$) in the simulated Mouse-Fibroblast-Sim dataset. Each data point represents an individual cell.

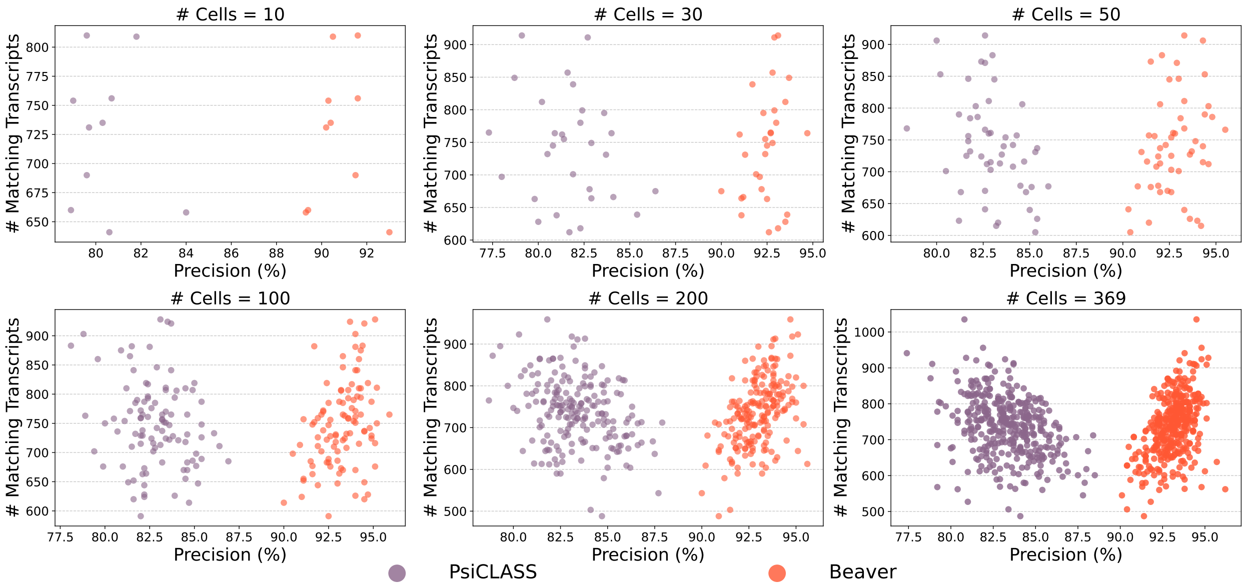

Supplementary Figure 17: Comparison of adjusted precision between Beaver and PsiCLASS across varying cell population sizes ($n = 10, 30, 50, 100, 200, 369$) in the simulated Mouse-Fibroblast-Sim dataset. Each data point represents an individual cell.

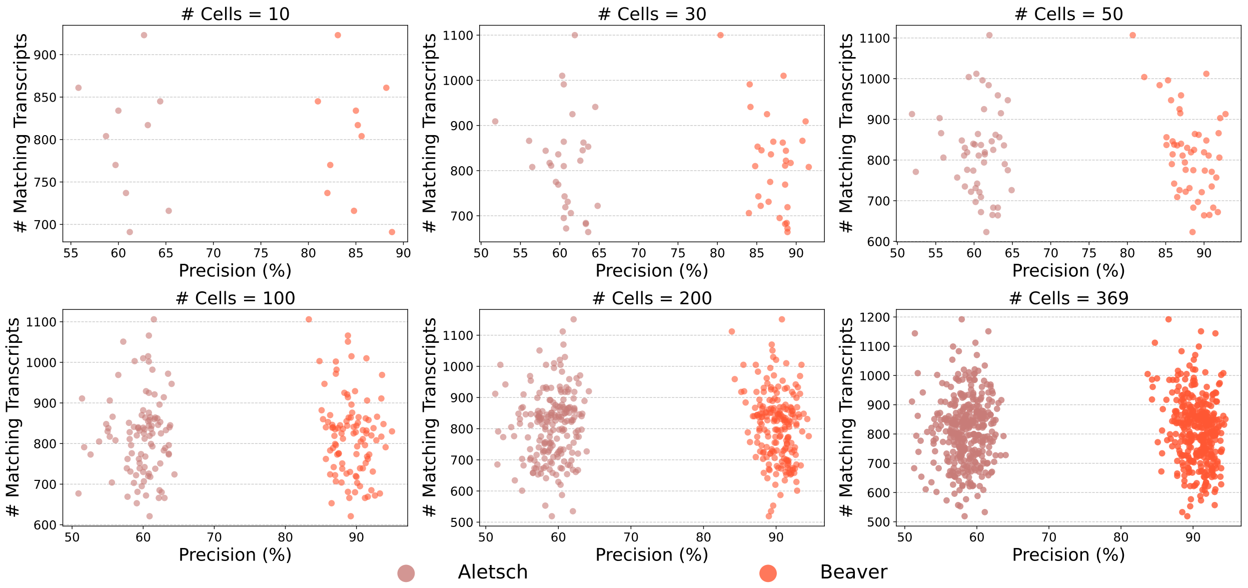

Supplementary Figure 18: Comparison of adjusted precision between Beaver and Aletsch across varying cell population sizes ($n = 10, 30, 50, 100, 200, 369$) in the simulated Mouse-Fibroblast-Sim dataset. Each data point represents an individual cell.

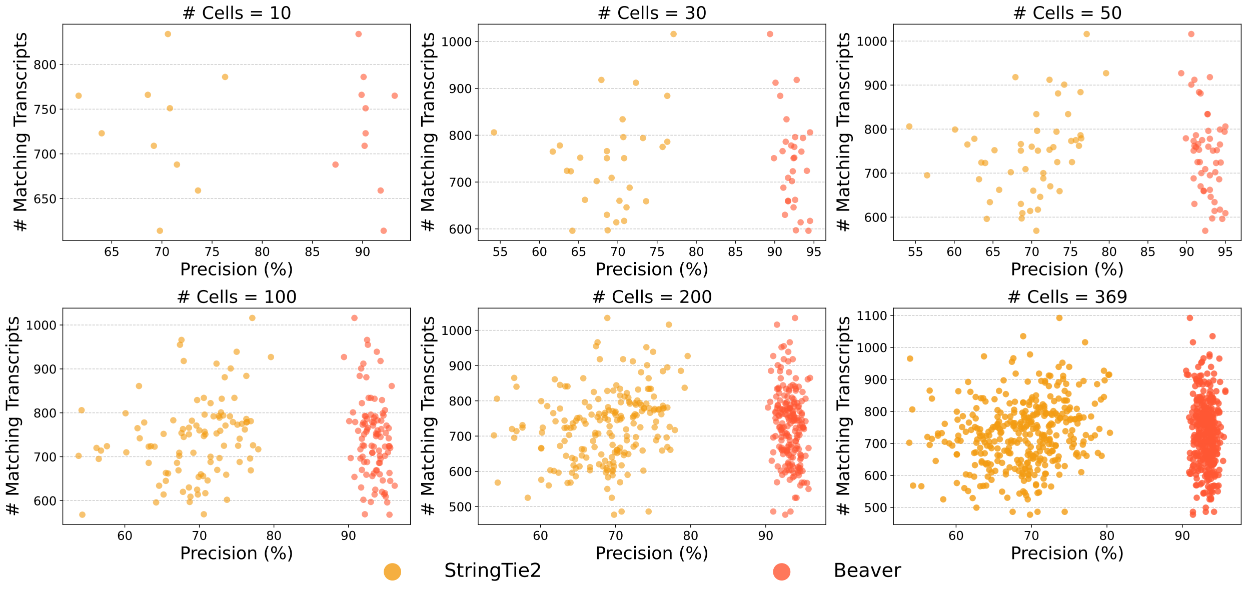

Supplementary Figure 19: Comparison of adjusted precision between Beaver and StringTie2 across varying cell population sizes ($n = 10, 30, 50, 100, 200, 369$) in the simulated Mouse-Fibroblast-Sim dataset. Each data point represents an individual cell.

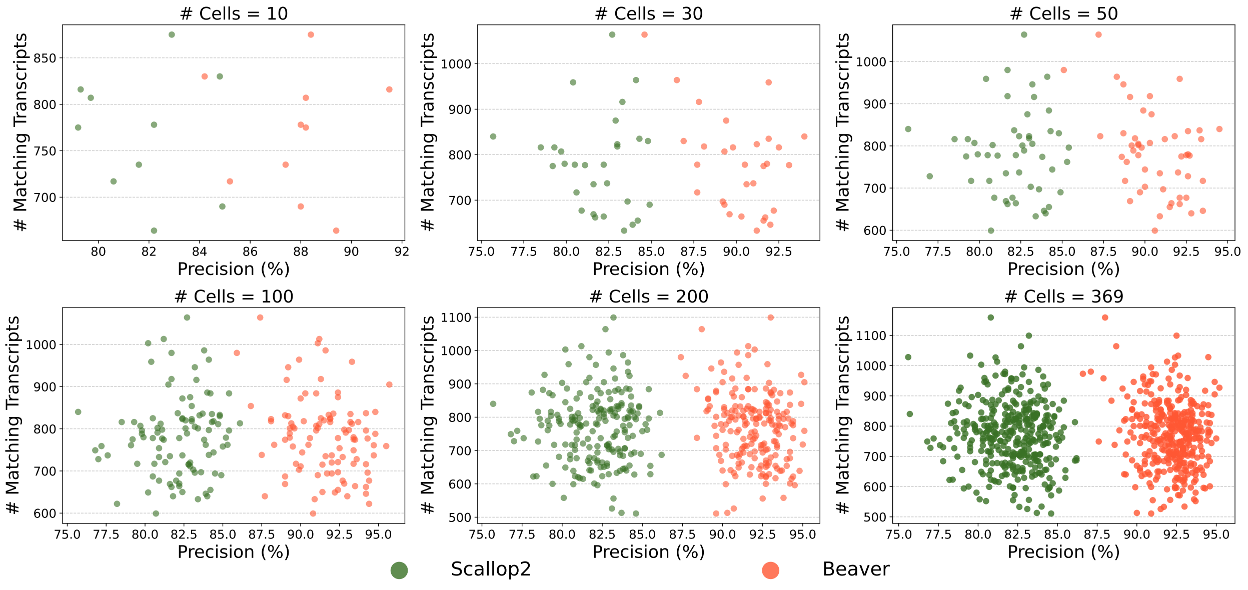

Supplementary Figure 20: Comparison of adjusted precision between Beaver and Scallop2 across varying cell population sizes ($n = 10, 30, 50, 100, 200, 369$) in the simulated Mouse-Fibroblast-Sim dataset. Each data point represents an individual cell.
